# *Egr-1*: A Candidate Transcription Factor Involved in Molecular Processes Underlying Time-Memory

**DOI:** 10.3389/fpsyg.2018.00865

**Published:** 2018-06-05

**Authors:** Aridni Shah, Rikesh Jain, Axel Brockmann

**Affiliations:** ^1^Tata Institute of Fundamental Research, National Centre for Biological Sciences, Bengaluru, India; ^2^School of Chemical and Biotechnology, SASTRA University, Thanjavur, India

**Keywords:** *Egr-1*, honey bee foraging, time-memory, anticipation, small Kenyon cells

## Abstract

In honey bees, continuous foraging is accompanied by a sustained up-regulation of the immediate early gene *Egr-1* (early growth response protein-1) and candidate downstream genes involved in learning and memory. Here, we present a series of feeder training experiments indicating that *Egr-1* expression is highly correlated with the time and duration of training even in the absence of the food reward. Foragers that were trained to visit a feeder over the whole day and then collected on a day without food presentation showed *Egr-1* up-regulation over the whole day with a peak expression around 14:00. When exposed to a time-restricted feeder presentation, either 2 h in the morning or 2 h in the evening, *Egr-1* expression in the brain was up-regulated only during the hours of training. Foragers that visited a feeder in the morning as well as in the evening showed two peaks of *Egr-1* expression. Finally, when we prevented time-trained foragers from leaving the colony using artificial rain, *Egr-1* expression in the brains was still slightly but significantly up-regulated around the time of feeder training. *In situ* hybridization studies showed that active foraging and time-training induced *Egr-1* up-regulation occurred in the same brain areas, preferentially the small Kenyon cells of the mushroom bodies and the antennal and optic lobes. Based on these findings we propose that foraging induced *Egr-1* expression can get regulated by the circadian clock after time-training over several days and *Egr-1* is a candidate transcription factor involved in molecular processes underlying time-memory.

## Introduction

Honey bee foraging has been one of the most fruitful behavioral paradigms in the study of sensory and cognitive capabilities of insects and animals in general (von Frisch, 1967; Giurfa, 2007; Chittka, 2017). Foragers continue to visit a highly rewarding food source for days and weeks till it gets exhausted. This persistent behavior enables researchers to train honey bee foragers to an artificial sugar-water feeder which then can be used as a tool for psychological experiments (Chittka et al., 1999; Wagner et al., 2013). For example, presenting the feeder at a specific time during the day showed that honey bees learnt the time of food presentation and demonstrated for the first time that animals have a sense of time (Beling, 1929; Wahl, 1932, 1933).

Since then, many behavioral studies followed, investigating foraging entrainment (= time of food presentation shifts behavioral/physiological rhythms which may/may not reflect a true time-place association) and time memory (= ability of individual foragers to associate the presence of food with both location and time of day) (Wahl, 1932; Koltermann, 1971; Moore et al., 1989; Naeger et al., 2011). Time-memory experiments showed that honey bee foragers are capable of associating food related cues like odor, color or spatial location with time (Gould, 1987; Zhang et al., 2006; Pahl et al., 2007; Prabhu and Cheng, 2008) and can memorize up to nine different feeder times per day (Koltermann, 1971). There is convincing evidence that daily foraging entrainment of bees and time-memory are regulated by the circadian clock (Renner, 1955, 1957, 1959; Beier, 1968; Frisch and Aschoff, 1987; Bloch, 2010; Fuchikawa et al., 2017).

Recently, we showed that continuous foraging is accompanied by a sustained up-regulation of the immediate early gene *Egr-1* (early growth response protein-1; see Chen et al., 2016; Duclot and Kabbaj, 2017) and candidate downstream genes involved in learning and memory (Singh et al., 2017). Our results indicated that up-regulation of *Egr-1* is dependent on the food reward. Now, we were interested in the question whether time-training over several days might affect the expression of *Egr-1*. Behaviorally, time-training of honey bee foragers leads to anticipatory activity (Moore et al., 1989; Moore and Doherty, 2009), thus it could be possible that time-training might also lead to an anticipatory molecular response. We performed a series of different time-training experiments similar to those that have been done before (Wahl, 1932; Moore et al., 1989; Naeger et al., 2011) but instead of testing the behavioral responses, we measured *Egr-1* expression on a test day at which the food reward was not presented.

Our experiments showed that *Egr-1* expression is highly correlated with the time and duration (hours) of feeder training even when the food reward is not presented. Foragers visiting a feeder over the whole day showed up-regulated *Egr-1* expression throughout the day, whereas foragers trained to visit a feeder for only a few hours in the morning or in the evening showed higher expression only during the respective training time. Foragers trained to visit two feeders at different times of the day showed two peaks of *Egr-1* up-regulation. Most importantly, foragers that were prevented from leaving the colony still showed a slight but significant up-regulation of *Egr-1* around the time of feeder training. These results suggest that *Egr-1* expression might get regulated by the circadian clock after time-training over several days. Up-regulation of *Egr-1* in the artificial rain experiments could be interpreted as an anticipatory molecular response. This conclusion is supported by the fact that the spatial expression pattern of *Egr-1* in the brain induced during foraging or activated in the artificial rain experiments, were very similar. We propose that *Egr-1* represents a candidate molecular link between the output of the circadian clock and the learning and memory systems involved in foraging.

## Materials and Methods

### Animal

*Apis mellifera* colonies were purchased from a local beekeeper and kept in an outdoor flight cage (12 m × 4 m × 4 m) on the campus of the National Centre for Biological Sciences, Bangalore, India. The colonies consisted of 4-frames within a standard commercial wooden hive box, each frame containing approximately 2500–3000 bees. The day-night length as well as the temperature conditions inside the flight cage were similar to the natural conditions in Bangalore with an approximate 12-h light-dark cycle all throughout the year. During the experimental period from November to February the time of sunrise changed from 06:15 to 06:45 and that of sunset changed from 17:50 to 18:30. The flight cage was devoid of any flora and the only food source available was the feeder provided by the experimenter.

### Training Regime

The training regime consisted of presentation of a colored plate with sugar-water solution (1 M) without odor, unless mentioned otherwise, for 10 days. We trained the bees for 10 days (a) since we need enough bees for the experiment (practical restriction) which can be achieved by more days of training since more bees are recruited (b) to increase temporal accuracy for the trained time, since Moore and Doherty (2009) show that with increase in the number of days, temporal accuracy increases. The duration and time of presentation differed according to the experiment. After the training time, the sugar-water was washed off and the plate kept back at the feeder location, to avoid association of the feeder plate with the food reward. Foragers trained to the feeder were marked at the feeder on the 7th day and the collections, as documented for each experiment below, were done on the 11th day.

### Collection Without Food Reward

Honey bee foragers were allowed to forage *ad libitum* (all day) or trained to forage in the morning (08:00 to 10:00) or in the evening (16:00 to 18:00). On the 11th day, the food reward was not added to the feeder plate. Marked foragers were collected at six different time-points at 4 h intervals: 06:00, 10:00, 14:00, 18:00, 22:00, and 02:00. Foragers were collected from the hive, which involved opening the hive and temporarily removing the comb frames from the hive. Each experiment was performed on a separate colony.

The bees were immediately flash frozen in liquid nitrogen and stored at -80°C until further processing for RNA isolation.

### Collection of Bees Trained to 2 Feeders

As a pilot experiment, we trained foragers to 2 different feeders within a day that were separated in space and time in December 2017. The colonies were placed in a flight cage that was longer, but had the same height and width (24 m × 4 m × 4 m). The food sources were placed at the opposite ends, almost 24 m apart from each other. One feeder was blue colored with 1 M sucrose scented with 20 ul Phenylacetaldehyde per liter of sucrose and the other feeder was green colored with 1 M sucrose scented with 20 ul Linalool per liter of sucrose. The blue feeder was opened in the morning, from 08:00 to 10:00 whereas the green feeder was opened in the evening from 16:00 to 18:00. The feeder plates were left in their position after the training time. On the 5th day of training, foragers coming to each feeder were individually marked with numbered tags. From the 7th day onward, observations were made, and the bees were classified into groups that continuously visited either the morning or evening or both the feeders.

On the 11th day, bees that visited both the feeders were collected at 06:00, 09:00, 13:00, 17:00, 22:00 and 02:00. The time-points were chosen such that the bees were caught at 60 min after the start of the feeder training for both the feeders (09:00 and 17:00) and at an intermediate time-point (13:00). The other time-points were chosen according to the single feeder experiment, “collection without food reward” mentioned above.

Bees that visited either the morning or the evening feeder, were collected only at the 2 trained time-points (09:00 and 17:00). Since many of the marked bees were lost by the 11th day, only three bees per time-point were successfully analyzed.

This experiment was repeated in February 2018 with the training duration reduced to 1 h, i.e., 08:00 to 09:00 in the morning and 17:00 to 18:00 in the evening, in order to increase the separation between the 2 feeder times. From the 8th day onward, foragers coming to each feeder were marked with paint marks on the thorax in the morning and the abdomen in the evening. Each day was marked with a different color. On the 11th day, collections were done as per the above-mentioned time-points. Marked foragers were collected from the hive, which involved opening the hive and temporarily removing the comb frames from the hive. Feeder visits of the bees shown in Supplementary Figure S2.

### Collection With “Artificial Rain” Setup

Honey bee foragers were trained to forage either in the morning (08:00 to 10:00), afternoon (12:00 to 14:00), or evening (16:00 to 18:00) during the months of December 2016, February 2017 and November 2017, respectively. On the 11th day, the “artificial rain” setup was started at 06:00. The “artificial rain” setup consisted of a box made of plexiglass which would hold water, called the water basin. The water flowed continuously through pores on the underside of the water basin. The entire setup was positioned such that the hive entrance was completely blocked by the falling water and hence prevented the bees from flying out. Any marked bee that crawled out of the hive and escaped, was caught and chilled on ice until the completion of the collections. Collections were made from 5 equidistant holes on the inner lid of the hive that was covered with a black chart paper. The holes had a flap cover which was opened at the time of collection and a 50 ml tube was placed over the hole. Bees that crawled up the tubes, being attracted to light, were chilled on ice and then the marked foragers were separated out and flash frozen. This collection method was adopted to prevent bees from flying out during collections. Since we were interested in the expression pattern in and around the trained time, we collected bees at half hour intervals starting from an hour before the trained time up until an hour after the trained time. The rest of the time-points corresponded to previous collection time-points.

Collection time-points for morning trained bees: 06:00, 07:00, 07:30, 08:00, 08:30, 09:00, 10:00, 14:00, 18:00, 22:00. Collection time-points for noon trained bees: 06:00, 10:00, 11:00, 11:30, 12:00, 12:30, 13:00, 14:00, 18:00, 22:00. Collection time-points for evening trained bees: 06:00, 10:00, 14:00, 15:00, 15:30, 16:00, 16:30, 17:00, 18:00, 22:00.

### Brain Dissection, RNA Isolation, cDNA Preparation and Quantitative PCR

Frozen brains were dissected on a dry ice platform in a glass cavity block in 100% ethanol. Brains were homogenized in TRIzol (Invitrogen, Life Technologies, Rockville, MD, United States) using a motorized homogenizer and RNA was extracted using the standard Trizol-chloroform method. Glycogen (20 mg/ml, Thermo Scientific, Life Technologies, Rockville, MD, United States) was added for increased recovery of RNA. cDNA was prepared using the SuperScript^TM^ III First-Strand Synthesis System (Invitrogen, Life Technologies, Rockville, MD, United States).

Primers for *Egr-1* and *RP49* qPCR were the same that were used in Singh et al. (2017). *Egr-1* primers recognise a region in exon 3, hence amplify all 3 isoforms of *Egr* reported by Sommerlandt et al. (2016). Primers for *Cry-2* were Forward: 5′-AGGTCTCACATACTCTTTACA-3′; Reverse: 5′-ACTGTTGGTACTGGTGGT-3′. The qPCR was performed following the same protocol as in Singh et al. using Kapa SybrGreen (KapaBiosystems, Wilmington, Massachusetts, United States). The standard curve method was followed and *RP49* was used as the internal control.

### cDNA Cloning

To generate riboprobes for *Egr-1*, primers (Forward: 5′-AAAGGGAGAGAGAGGATGAAG-3′; Reverse: 5′-TAATGCGGTGGTGTGAGTTC-3′) were generated to amplify a 1096 bp fragment of the gene in exon 3. RNA was isolated and converted to cDNA following the procedure as described above and the cDNA was used as a template to amplify the fragment. The fragment was then purified using PCR Purification kit (Qiagen, Hilden, Germany) followed by cloning of the fragment into the pCR^TM^II-TOPO^®^ vector using the TOPO TA Cloning Kit (Invitrogen, Life Technologies, Rockville, MD, United States) following the manufacturer’s protocol. The cloning mix was transformed into *E. coli* (DH5-alpha) and screened using the blue-white screening regime. The plasmids were then isolated and sequenced for confirming the presence and orientation of insert.

### RNA *in Situ* Hybridization

Time-trained “active foragers” were collected from the feeder at 60 min after the onset of foraging. Time-trained “non-active foragers” were caught at 60 min after onset of the training time from the hive using the “artificial rain” setup. Time-trained control bees were caught at 6 h before the trained time from the hive. The bee brains were freshly dissected on DEPC water and immediately embedded into the Jung Tissue Freezing Medium (Leica Microsystems, Nussloch, Germany). The embedded brains were then sectioned using a HYRAX C-25 cryostat into 12 μm thin sections and collected on Superfrost Plus Microscope slides (Fisherbrand, Hampton, NH, United States). The slides were allowed to dry at room temperature for about 10 min and kept on dry ice until further processing.

RNA probes were synthesized using SP6 Polymerase or T7 Polymerase using DIG RNA labeling mix (Roche, Indianapolis, IN, United States) incubated for 2 h at 37°C. The probes were then purified using the Qiagen Micro kit (Qiagen, Hilden, Germany) and stored at -80°C.

The slides were fixed in 4% PFA overnight at 4°C. The slides were washed for 20 min in 0.1 M phosphate buffer (PB), followed by treatment with 10 mg/ml Proteinase K solution for 15 min at room temperature (RT), re-fixation in 4% PFA for 15 min at 4°C, followed by treatment with 0.2 M HCl for 10 min and 0.25% acetic anhydride in TEA for 10 min. Each step was followed by a 5 min wash with 0.1 M PB. The slides were dehydrated through an ethanol gradient of 70% → 95% → 100% and air-dried for 1 h. The slides were pre-hybridized in the hybridization buffer (50% formamide, 10 mM Tris–HCl pH 7.6, 200 ug/ml tRNA, 1X Denhardt solution, 10% Dextran sulfate, 600 mM NaCl, 0.25% SDS, 1 mM EDTA) without riboprobes for 1 h at 60°C. The riboprobes were added to the hybridization buffer followed by denaturation at 85°C for 5 min. The denatured probes were added to the slides and allowed to hybridize overnight at 60°C in a mineral oil bath.

The slides were then washed through a series of SSC buffer, starting with 5X SSC (rinse), 1:1 solution of formamide and 2X SSC for 30 min at 60°C, followed by 2X SSC and 0.2X SSC for 20 min each at 60°C and finally, 3 washes with TNT (0.1 M Tris, 0.15 M NaCl, 0.05% Tween) at RT.

For detection, the slides were first blocked with 5% BSA for 30 min at RT, followed by incubation in Anti-DIG POD (Roche, Indianapolis, IN, United States) overnight at 4°C. After incubation, slides were washed in TNT and incubated in Tyramide-Cy5 (Perkin Elmer, MA, United States) for 15 min followed by washing and mounting with Vectashield with DAPI (Vector Laboratories, CA, United States). The fluorescent images were captured using Olympus FV1000 at a magnification of 10 × with 1 um thick optical sections. *Post hoc* adjustments of brightness and intensity were made using ImageJ analysis software (NIH, United States).

### Statistics

All statistical analyses were performed using R [R 3.4.1 GUI 1.70 El Capitan build (7375)] (R Core Team, 2017). Since the data-points were not normally distributed, Kruskal Wallis (KW) tests were done. When the KW-test was significant, *post hoc* analyses for comparison amongst the groups was done using the dunn.test package in R (Dinno, 2017) with *p*-values adjusted for multiple comparisons using the Benjamini-Hochberg (“bh”) method (Benjamini and Hochberg, 1995). The alpha was set at 0.05. All data are represented as box-plots with individual data-points indicated.

## Results

### Restricted Time-Training Leads to Time-Restricted *Egr-1* Up-Regulation

**FIGURE 1:**
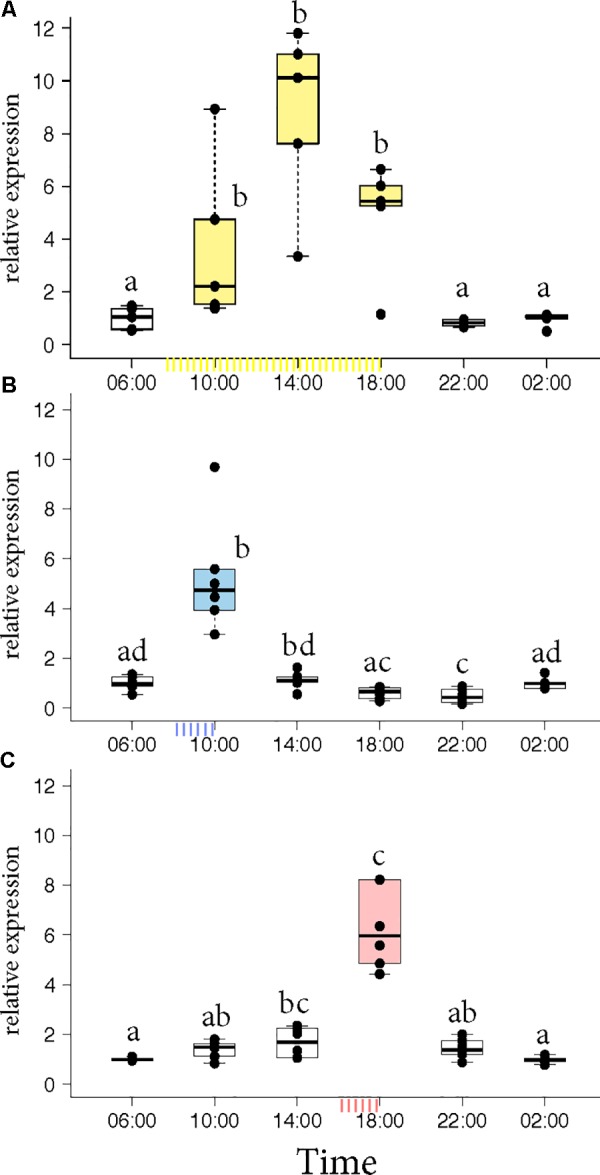
*Egr-1* expression in the absence of food reward. **(A)** *Ad libitum* (yellow) fed bees show elevated levels of *Egr-1* all through the day, with a peak at 14:00. **(B,C)** Morning trained (blue) and evening trained (red) bees showed higher expression only at the trained time and significantly lower levels at all other time-points. Data shown as relative expression changes compared to 06:00 in the form of box-plots with individual data-points delineated, *n* = 6. KW-test with Dunn’s (“bh” method) multiple comparison was done on each experiment, *p*-values are shown in Supplementary Tables S1–S3, respectively.

Honey bee foragers were allowed to forage *ad libitum* or were trained either to a morning feeder (08:00 to 10:00) or to an evening feeder (16:00 to 18:00) for 10 days. On the 11th day, the marked foragers were collected from the hive in the absence of food reward. Honey bees that foraged at the *ad libitum* feeder showed elevated expression of *Egr-1* throughout the day with highest expression at 14:00 (**Figure 1A**, *p*-values in Supplementary Table S1). Foragers that were trained to a morning or an evening feeder showed significant up-regulation in the mRNA levels of *Egr-1* at the time of feeder training, i.e., 10:00 in the morning trained and 18:00 in the evening trained foragers compared to most of the other time-points (**Figures 1B,C**, *p*-values in Supplementary Tables S2, S3). The time point directly following the training time in the morning experiment and the one preceding the training time in the evening experiment showed *p*-values that were slightly above the cut off (morning 14:00: *p* = 0.06; evening 14:00 *p* = 0.07). Restricted foraging for a short time of the day led to a restricted *Egr-1* expression occurring only around the time of training.

### Individual Foragers Trained to 2 Feeders at Different Times of the Day Showed 2 Peaks of *Egr-1* Expression

Next, we tested the dynamics of *Egr-1* expression in individual bees trained to 2 different feeders separated in space and time. Both feeders differed in color and odor and one was opened in the morning while the other was opened in the evening. Presenting a colony with 2 feeders at different times of the day resulted in 3 foraging groups: (a) bees that visited only the morning feeder (“only morning”), (b) bees that visited only the evening feeder (“only evening”), and (c) bees that visited both the feeders (“both feeder”).

**FIGURE 2:**
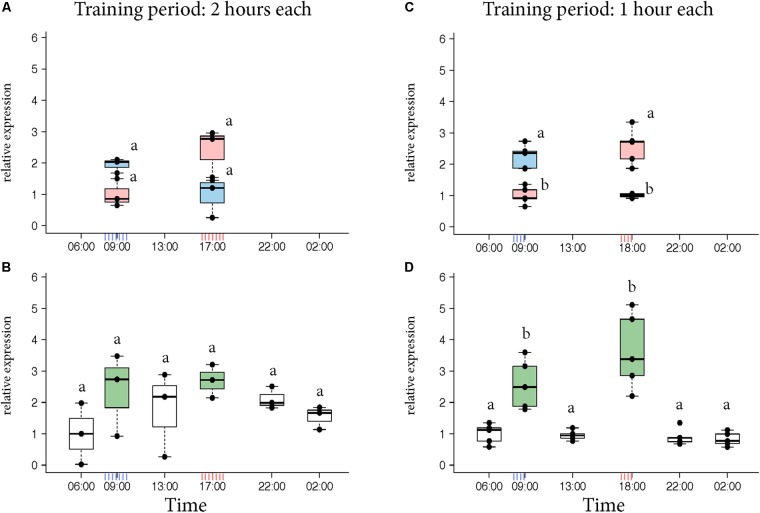
*Egr-1* expression in individuals exposed to two feeders. **(A,B)** Bees were trained for 2 h each in the morning and evening. **(A)** Those bees that visited only the morning feeder (blue) or the evening feeder (red) showed comparatively higher expression in the morning or evening, respectively, however, not significant. **(B)** Bees that visited both the feeders (green) showed comparatively higher expression at both the time-points. The time-point in between the 2 training times (13:00) showed a down-regulation trend, however, none of the time-points were significantly different. *n* = 3 per time-point since enough bees could not be caught. **(C,D)** Experiment was repeated with 1-h training period each to increase separation between the training times. **(C)** Bees that visited the morning (blue) feeder showed significantly higher expression at 09:00 compared to the “morning only” bees at 18:00 as well as “evening only” bees at 09:00. Similarly, “evening only” (red) bees showed significantly higher expression at 18:00 compared to “evening only” bees at 09:00 as well as “morning only” bees at 18:00. **(D)** Bees that visited both feeders (green) showed significantly higher expression at both the trained time-points compared to all other time-points. 13:00 showed significantly lower levels of *Egr-1*; *n* = 5 per time-point. Data shown as relative expression changes compared to the lowest value per group in the form of box-plots with individual data-points delineated. KW-test with Dunn’s (“bh” method) multiple comparison was done for single feeder visiting bees (“only morning” + “only evening”) and “both feeder” visiting bees, *p*-values are shown in Supplementary Tables S4, S5, respectively.

*Egr-1* brain expression levels of the bees that visited only one feeder showed a peak at the time they had been trained to visit the feeder similar to our previous experiments (**Figure 2A**, KW test: ns; **Figure 2C**, *p*-values in Supplementary Table S4). In contrast, *Egr-1* expression levels of the bees that visited both feeders showed two peaks, one at each training time (**Figure 2B**, KW test: ns; **Figure 2D**, *p*-values in Supplementary Table S5). When the bees were trained to visit the feeders for only 1 h starting at 08:00 and 17:00, expression of *Egr-1* was significantly down-regulated at the intermediary time-point of 13:00 (**Figure 2D**). In the 2 h training experiment, the *Egr-1* expression was not down regulated at 13:00 (**Figure 2B**). A greater temporal separation of the training period led to a more distinct regulation of *Egr-1* expression.

### Foragers Visiting “Both Feeders” Showed *Cry-2* Expression Similar to “Evening Only” Bees

**FIGURE 3:**
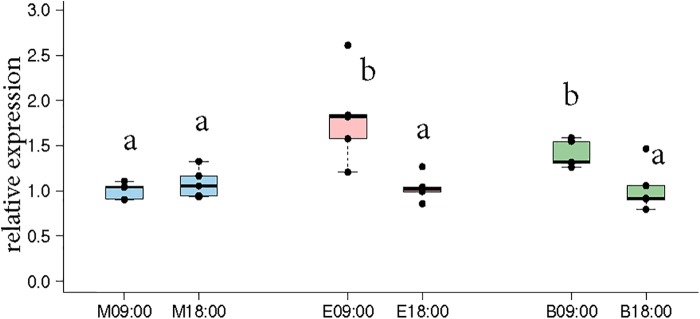
*Cry-2* expression comparison between morning time-point (09:00) and evening time-point (18:00) in the same bees as shown in **Figures 2C,D**. “Morning only” (M09:00, M18:00; blue) bees showed no significant differences in *Cry-2* expression whereas “evening only” (E09:00, E18:00; red) bees showed significantly higher expression in the morning compared to evening. “Both feeder” (B09:00, B18:00; green) bees showed significantly higher expression in the morning compared to evening, similar to “evening only” bees. The lower expression value in all 3 groups were not significantly different from each other. Data shown as relative expression changes compared to the lowest value in the form of box-plots with individual data-points delineated. KW-test with Dunn’s (“bh” method) multiple comparison was done on the entire data-set, *p*-values are shown in Supplementary Tables S4, S5, respectively.

Naeger et al. (2011) showed that morning and evening trained foragers differ in the expression pattern of *Cry2* and *Per* indicating that the foragers likely developed different circadian rhythms according to their foraging activity. Therefore, we got interested in the question how foragers visiting one or two feeders over the day differ in *Cry2* expression. In the “morning only” bees, *Cry-2* expression levels at 09:00 and 18:00 were very similar, whereas in the “evening only” bees, *Cry-2* expression levels were significantly higher at 09:00 compared to 18:00 (**Figure 3**, *p*-values in Supplementary Table S6). These results are consistent with those of Naeger et al. (2011) who showed a similar expression pattern for *Cry-2*, when they trained bees from 09:00 to 10:15 or from 17:00 to 18:15 and looked at transcripts levels at the 2 trained time-points for both the groups. The bees that visited “both feeders” in our experiments showed a *Cry2* expression pattern similar to the “evening only” foragers, with significantly higher expression at 09:00 compared to 18:00.

Different to the earlier experiments, in which the training time was forced onto the bees, bees in our experiments could choose when to forage and this decision then influenced their circadian clock.

### *Egr-1* Got Up-Regulated in Time-Trained Foragers Prevented From Flying Out

To test whether *Egr-1* expression in time-trained foragers is regulated by the circadian clock, we tested *Egr-1* expression in time-trained foragers that were prevented from flying out. If *Egr-1* is under the influence of the circadian clock, it would be up-regulated at the trained time even in the absence of flight activity and other environmental cues. To reduce any stress responses that might occur in bees mechanically restricted from leaving the colony, we used an “artificial rain” setup (Supplementary Figure S1A) (Riessberger and Crailsheim, 1997). As in the case of natural rain, the foragers would not fly out. The feeder-trained foragers were collected from the hive.

**FIGURE 4:**
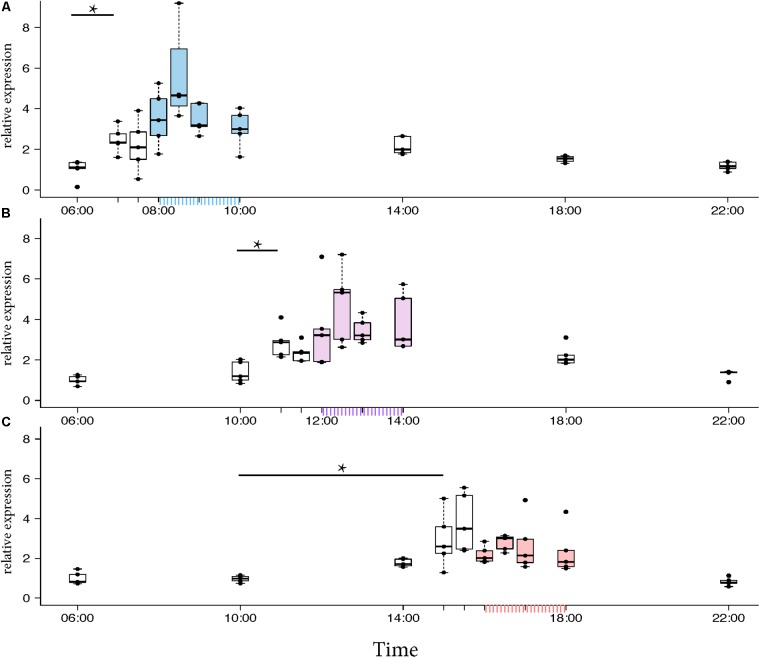
*Egr-1* expression when the bees were prevented from flying out using “artificial rain” setup. **(A)** Bees that were trained from 08:00 to 10:00, already showed significant up-regulation of *Egr-1* by 07:00 and remained up-regulated till the end of training time with a peak at 08:30. The mRNA levels started to decline at 09:00 and was reduced significantly by 14:00 and remained low for the rest of the day. **(B)** Bees that were trained from 12:00 to 14:00, showed significant up-regulation by 11:00 with a peak at 12:30. The expression declined thereafter and was significantly low by 18:00. **(C)** Bees that were trained from 16:00 to 18:00 showed an up-regulation trend already by 14:00, although not significant. mRNA levels were significantly increased by 15:00 with a peak at 15:30 which started to decline thereafter, differing from the trends seen in morning and afternoon trained bees. Data shown as relative expression changes compared to 06:00 in the form of box-plots with individual data-points delineated, *n* = 5. KW-test with Dunn’s (“bh” method) multiple comparison was done on each experiment, *p*-values are shown in **Tables 1**–**3**, respectively.

Morning trained foragers prevented from flying out showed a slight but significant up-regulation of *Egr-1* about an hour before the trained time, i.e., at 07:00 and the up-regulation was maintained till the end of training time with a peak at 08:30. The expression levels dropped after the trained time, and at 18:00 the expression was significantly lower compared to the highest level of *Egr-1* expression i.e., 08:30 and hence had dropped to levels equivalent to 06:00 (**Figure 4A**, *p*-values in **Table 1**).

**Table 1:** Adjusted *p*-values for Artificial Rain Experiment (08:00-10:00 trained).

	10:00	14:00	18:00	22:00	06:00	07:00	07:30	08:00	08:30
**14:00**	0.21								
**18:00**	0.06	0.21							
**22:00**	**0.0127**	0.08	0.30						
**06:00**	**0.0093**	0.06	0.27	0.46					
**07:00**	0.30	0.39	0.15	0.05	**0.0414**				
**07:30**	0.20	0.46	0.23	0.10	0.08	0.36			
**08:00**	0.39	0.15	**0.0369**	**0.0059**	**0.0055**	0.22	0.13		
**08:30**	0.16	**0.0402**	**0.0061**	**0.0010**	**0.0011**	0.07	**0.0386**	0.23	
**09:00**	0.37	0.13	**0.0352**	**0.0051**	**0.0053**	0.20	0.11	0.46	0.25

*p-values less than 0.05 shown in bold*.

In the afternoon trained foragers, *Egr-1* showed an expression pattern similar to morning trained foragers with significant elevation at 11:00 compared to the 10:00 and 06:00. The elevated expression was maintained till the end of training time with a peak at 12:30 and then dropped significantly by 18:00 (**Figure 4B**, *p*-values in **Table 2**).

**Table 2:** Adjusted *p*-values for Artificial Rain Experiment (12:00-14:00 trained).

	10:00	11:00	11:30	12:00	12:30	13:00	14:00	18:00	22:00
**11:00**	**0.0400**								
**11:30**	0.10	0.34							
**12:00**	**0.0312**	0.45	0.31						
**12:30**	**0.0047**	0.21	0.10	0.26					
**13:00**	**0.0085**	0.31	0.16	0.34	0.39				
**14:00**	**0.0088**	0.32	0.17	0.35	0.39	0.48			
**18:00**	0.18	0.25	0.39	0.20	0.05	0.10	0.10		
**22:00**	0.45	**0.0326**	0.09	**0.0236**	**0.0037**	**0.0083**	**0.0083**	0.16	
**06:00**	0.34	**0.0098**	**0.0382**	**0.0078**	**0.0021**	**0.0042**	**0.0033**	0.07	0.35

*p-values less than 0.05 shown in bold*.

In the evening trained foragers, the *Egr-1* expression was very low in the morning, with no difference in levels at 06:00, 10:00 and 22:00. An up-regulation trend was observed at 14:00, however, it was not statistically significant. A statistically significant up-regulation was observed at 15:00_,_ and the up-regulation was maintained till the end of training time with a peak at 15:30. The expression levels dropped down to minimum values at 22:00 (**Figure 4C**, *p*-values in **Table 3**; Supplementary Figure S1B, *p*-values in Supplementary Table S7).

**Table 3:** Adjusted *p*-values for Artificial Rain Experiment (16:00-18:00 trained).

	10:00	14:00	15:00	15:30	16:00	16:30	17:00	18:00	22:00
**14:00**	0.12								
**15:00**	**0.0105**	0.16							
**15:30**	**0.0020**	**0.0419**	0.31						
**16:00**	**0.0302**	0.30	0.35	0.16					
**16:30**	**0.0043**	0.09	0.41	0.42	0.26				
**17:00**	**0.0232**	0.26	0.40	0.20	0.45	0.30			
**18:00**	**0.0419**	0.35	0.30	0.12	0.44	0.20	0.40		
**22:00**	0.40	0.07	**0.0049**	**0.0018**	**0.0189**	**0.0021**	**0.0124**	**0.0243**	
**06:00**	0.50	0.12	**0.0118**	**0.0030**	**0.0322**	**0.0051**	**0.0251**	**0.0444**	0.41

*p-values less than 0.05 shown in bold*.

Together, our artificial rain experiments clearly showed that *Egr-1* expression is up-regulated in time-trained foragers without any foraging or flight activity. This molecular response resembles anticipatory behavior of time-trained honey bee foragers (Moore et al., 1989).

### “Active Foragers” and Time-Trained “Non-active Foragers” Show *Egr-1* Expression in the Same Population of Mushroom Body Cells (Small Kenyon Cells)

To identify the brain regions that could be involved in foraging and time-training related *Egr-1* up-regulation, we performed brain *in situ* hybridization for *Egr-1*. Specifically, we compared the *Egr-1* expression pattern of brains from actively foraging honey bees caught 60 min after the onset of foraging (“active foragers”) (see Singh et al., 2017) and time-trained but not flying foragers caught 60 min after onset of the training time (“non-active foragers”). As a control we used foragers caught from the hive, 6 h before the training time.

**FIGURE 5:**
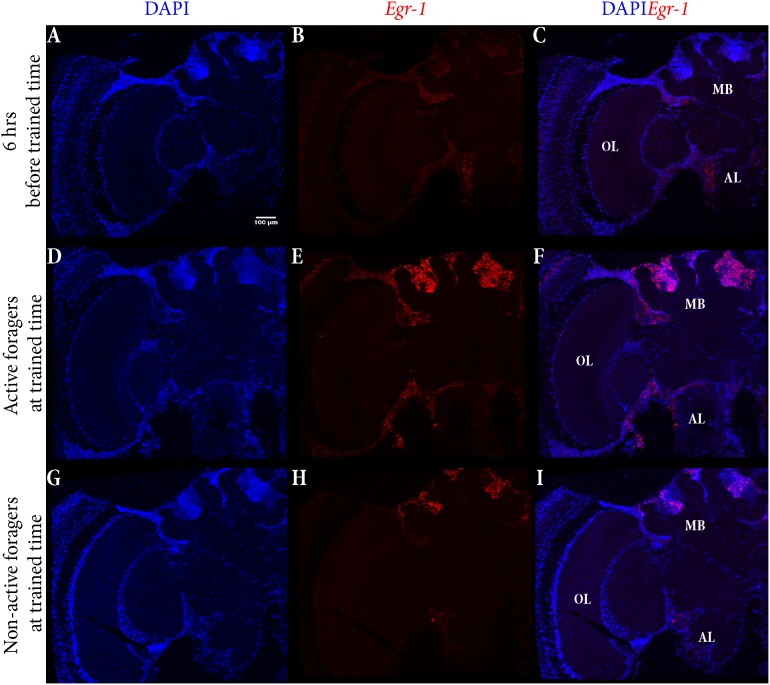
*In situ* hybridization of *Egr-1* on brains of foragers. **(A–C)** Trained bees that were collected 6 h before the trained time from the hive showed very low expression of *Egr-1*, with only a few cells in the antennal lobes stained. **(D–F)** “Active foragers”, collected from the feeder at 60 mins past the start of foraging time, showed strong *Egr-1* expression in the mushroom bodies as well as other brains parts like antennal lobes and optic lobes. **(G–I)** Time-trained “non-active foragers,” collected from the hive with the “artificial rain”setup at 60 min past the trained time, showed specific expression only in the small Kenyon cells. MB, mushroom bodies; OL, optic lobes; AL, antennal lobes.

**FIGURE 6:**
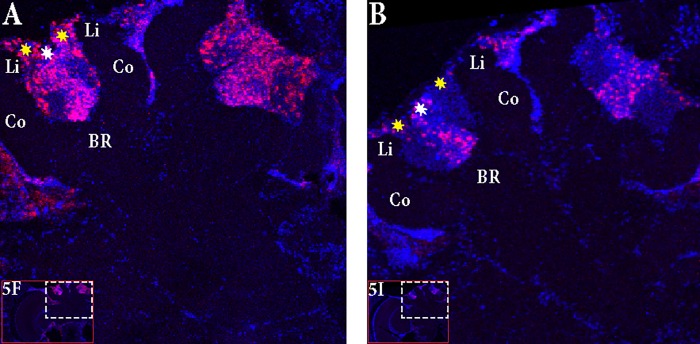
Focus on the mushroom bodies of the “active foragers” and the “non-active foragers”. **(A)** Almost all the small Kenyon cells (white stars) are stained for *Egr-1* whereas only some of the large Kenyon cells (yellow stars) that are closer to the calyces show staining in the “active foragers”. **(B)** “Non-active foragers” showed very specific staining of the small Kenyon cells (white stars) and a few cells close to the “lip” region of the calyces only. Li, Lip; Co, Collar; BR, Basal Ring.

The control bees showed very low staining with only a few cells in the antennal lobes stained (**Figures 5A–C**). In “active foragers”, predominant expression of *Egr-1* was seen in the small Kenyon cells (sKCs) compared to large Kenyon cells (lKCs), where only few cells showed staining (**Figures 5D–F**, **6A**). “Non-active foragers” also showed *Egr-1* expression in the sKCs. The expression was lower compared to “active foragers” and more specifically expressed in the sKCs. Very few lKCs were stained in the “non-active foragers” suggesting anticipatory up-regulation of *Egr-1* specifically in the sKCs (**Figures 5G–I**, **6B**). We limited our analysis to the mushroom bodies, because they allow a clear identification and comparison of neuron populations between different individuals. Our stainings suggest that there might be additional neuron populations in other brain areas involved in these processes.

## Discussion

The major finding of our study is that time-restricted foraging and feeder time-training over several days led to time-restricted *Egr-1* daily expression pattern. Foragers that visited one feeder for a restricted time period showed one peak of *Egr-1* expression, whereas those that visited two different feeders at two separate times of the day showed highest expression at the 2 trained time-points. Even more importantly, time-trained foragers that were prevented from flying out showed significant *Egr-1* expression around the time of training indicating that training time is sufficient to induce *Egr-1* up-regulation. These experiments suggest that bees respond to time-training not only with anticipatory behavior but also an anticipatory molecular response. *Egr-1* is already slightly up-regulated in expectation of a food reward.

Based on these and earlier results, we propose that *Egr-1* expression is regulated by foraging associated food reward as well as the circadian clock after several days of time-training. We cannot comment upon acquisition of memory or the expression profile of *Egr-1* in the initial days of training since we have not tested it. It is possible that *Egr-1* is up-regulated after a single day of training but the temporal accuracy of expression might be affected similar to the anticipatory behavior (Moore and Doherty, 2009).

In “active foragers,” *Egr-1* is expressed in the cells of the mushroom bodies (MB), optic lobes (OL), and antennal lobes (AL). Since MBs are thought to be involved in learning and memory processes (Mizunami et al., 1998; Hourcade et al., 2010; Lefer et al., 2012), we focused for now, on the expression in the MBs. Within the MBs, the small Kenyon cells (sKCs) showed predominant staining whereas only some of the lKCs closer to the calyces showed *Egr-1* expression (**Figure 6A**). In “non-active foragers,” *Egr-1* was expressed in the sKCs only (**Figure 6B**). Therefore, the sKCs may play a crucial role in foraging related time-memory. Interestingly, some of the candidate downstream targets of *Egr-1* (Khamis et al., 2015) that showed significant expression during foraging (Singh et al., 2017), namely, *Hr38* (Yamazaki et al., 2006), *EcR* (Takeuchi et al., 2007), and *DopR2* (McQuillan et al., 2012) have been shown to be specifically expressed in the sKCs.

Although there is some information about differences in developmental origin, sensory inputs as well as the expression of particular genes between the different Kenyon cell types, we do not know anything about their functions and a functional separation among them.

The sKCs form a central cluster directly located above the basal ring, which their dendrites innervate. The basal ring receives multiple sensory inputs from optic lobes, in particular the lobula, (Ehmer and Gronenberg, 2002), antennal lobes (Gronenberg, 2001), and the suboesophageal ganglia (Schröter and Menzel, 2003). Farris et al. (1999) showed that the sKCs are the last Kenyon cells to be generated during development and proposed that they might be involved in the MB growths at the nurse-forager transition.

The lKCs are separated in a central cluster that innervates the collar and an outer cluster that innervates the lip region of the calyces. The collar receives inputs only from the visual system and the lip only from the olfactory system (Strausfeld, 2002; Farris, 2013). Given the differences in the sensory inputs, it is tempting to speculate that the sKCs might have a unique function in foraging related, i.e., food reward induced, learning processes and time-memory. In contrast, Lutz and Robinson (2013) reported a pronounced *Egr-1* expression in the lKCs during orientation flights, which precede foraging and are independent of food reward.

The expression pattern of *Cry2* showed that “morning only” and “evening only” foragers have different *Cry2* expression patterns, suggesting that they are on different circadian time schedules. Bees foraging at “both feeders” in the morning and the evening showed an expression pattern similar to “evening only” bees although they were foraging in the morning and the afternoon. So far, bee chronobiologists have made a distinction between entrainment and non-entrainment time memory models (Moore, 2001). The entrainment model proposes that the clock oscillator will get entrained to the time of the food presentation which then shifts behavioral or physiological rhythms similar to changes in the light/dark cycle. The non-entrainment model hypothesizes that a representation of the circadian phase at which a foraging experience occurred is stored together with features of the food source in a separate memory system (Moore and Doherty, 2009). Both mechanisms might not necessarily exclude each other and could act in parallel (Mistlberger, 1994). The results of our 2-feeder experiment actually support the idea, that both processes might be intertwined. Foraging entrainment affects the cycling of clock genes (master oscillator), and time-memory could be based on an association of *Egr-1* expression and a specific phase of the clock cycling (memory of oscillator phase).

Frisch and Aschoff (1987) clearly demonstrated that time-restricted feeder presentation under constant light/dark cycle leads to an entrainment of a colonies’ foraging activity. So far nothing is known about the sensory channel and respective clock neurons in the brain involved in this foraging entrainment. There are two plausible mechanisms, either foraging entrainment is based on an independent food entrainable oscillator (FEO) or foraging entrainment modulates some part of the canonical light entrainable oscillator (LEO) master clock. As honey bees are dependent on a time-compensated sun compass for navigation, information of the light/dark cycle is highly likely present in foraging entrained foragers.

The artificial rain experiments demonstrated that *Egr-1* expression is initiated in time-trained foragers at least an hour before the training time. In the evening trained foragers, this up-regulation trend appears to start 2 h before the trained time, although not significant. These expression patterns fit with previous work on anticipatory flight behavior that demonstrated that bees trained in the morning and afternoon show shorter anticipatory flight activity whereas those trained in the evening show longer anticipatory flight activity (Moore et al., 1989). Therefore, *Egr-1* could be a molecular equivalent of anticipatory behavior.

**FIGURE 7:**
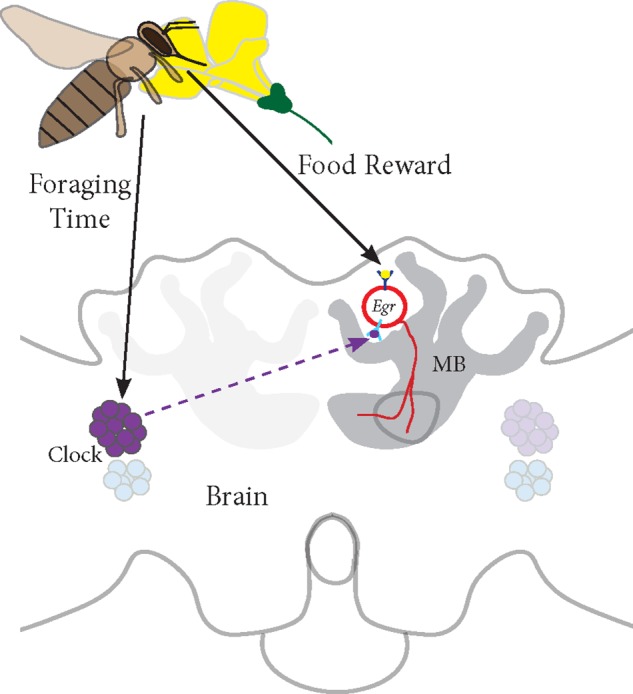
Proposed model for *Egr-1* function in honey bee foraging: (a) Foraging/food reward leads to an up-regulation of *Egr-1* in the sKCs, which in turn regulates the expression of downstream targets that are involved in learning and memory. (b) Time-Restricted foraging at one food source leads to entrainment of the molecular clock. This effect might be restricted to a specific population of clock cells. For example, different populations of clock cells might be involved in foraging entrainment and time-compensated sun compass navigation. (c) Time-training over several days leads to an anticipatory up-regulation of *Egr-1* that is controlled by the circadian clock. Thus, Egr-1 expression in the Kenyon cells of the mushroom bodies might be regulated via two signaling mechanisms, one from food reward related pathways and one from the circadian clock.

Based on our studies, we propose a model for *Egr-1* function in honey bee foraging (**Figure 7**):

(a) Foraging/food reward leads to an up-regulation of *Egr-1* in the sKCs, which in turn regulates the expression of downstream targets that are involved in learning and memory.
(b) Time-restricted foraging in the morning or afternoon (Naeger et al., 2011) or at both the time-points leads to a change in the expression of the clock genes. This effect might be restricted to a specific population of clock cells such that the information of the light/dark cycle is still retained in the remaining clock cells.
(c) Time-training over several days leads to an anticipatory up-regulation of *Egr-1* that is controlled by the circadian clock. Thus, *Egr-1* expression in the Kenyon cells of the mushroom bodies might be regulated via two signaling mechanisms, one from food reward related pathways and one from the circadian clock. This signal molecule of the circadian clock could be PDF (peptide dispersing factor) which is the commonly known signaling molecule produced by the clock cells. Detailed study of PDF expressing neurons in honey bees show that the network of PDF-positive fibers extends extremely close to the calyces of the mushroom bodies but does not enter them. Additionally, they show that the level of PDF oscillates in these neurites in a daily manner (Beer et al., 2018). Therefore, PDF could be a potential candidate for foraging-related time communication.

## Author Contributions

AS and AB designed the experiments of the study and wrote the manuscript. AS and RJ performed the experiments and analyzed the data.

## Conflict of Interest Statement

The authors declare that the research was conducted in the absence of any commercial or financial relationships that could be construed as a potential conflict of interest.

## References

[B1] Beer K. Kolbe E. Kahana N. B. Yayon N. Weiss R. Menegazzi P. (2018). Pigment-Dispersing factor-expressing neurons convey circadian information in the honey bee brain. *Open Biol.* 8:170224. 10.1098/rsob.170224 29321240 PMC5795053

[B2] Beier W. (1968). Beeinflussung der inneren Uhr der Bienen durch Phasenverschiebung des Licht–Dunkel–Zeitgebers. *Zeitschrift Bienenforschung* 9 356–378.

[B3] Beling I. (1929). Über das Zeitgedächtnis der Bienen. *Z. Vergl. Physiol.* 9 259–338. 10.1007/BF00340159

[B4] Benjamini Y. Hochberg Y. (1995). Controlling the false discovery rate: a practical and powerful approach to multiple testing. *J. R. Stat. Soc. Series B* 57 289–300. 10.2307/2346101

[B5] Bloch G. (2010). The social clock of the honeybee. *J. Biol. Rhythms* 25 307–317. 10.1177/0748730410380149 20876811

[B6] Chen X. Rahman R. Guo F. Rosbash M. (2016). Genome-wide identification of neuronal activity-regulated genes in *Drosophila*. *eLife* 5:e19942. 10.7554/eLife.19942.001 27936378 PMC5148613

[B7] Chittka L. (2017). Bee cognition. *Curr. Biol.* 27 R1049–R1053. 10.1016/J.CUB.2017.08.008 29017035

[B8] Chittka L. Thomson J. D. Waser N. M. (1999). Flower constancy, insect psychology, and plant evolution. *Naturwissenschaften* 86 361–377. 10.1007/s001140050636

[B9] Dinno A. (2017). *dunn.test: Dunn’s Test of Multiple Comparisons Using Rank Sums. R package version 1.3.5.* Available at: https://cran.r-project.org/ web/packages/dunn.test/index.html

[B10] Duclot F. Kabbaj M. (2017). The role of early growth response 1 (EGR1) in brain plasticity and neuropsychiatric disorders. *Front. Behav. Neurosci.* 11:35. 10.3389/fnbeh.2017.00035 28321184 PMC5337695

[B11] Ehmer B. Gronenberg W. (2002). Segregation of visual input to the mushroom bodies in the honeybee (*Apis mellifera*). *J. Comp. Neurol.* 451 362–373. 10.1002/cne.10355 12210130

[B12] Farris S. M. (2013). Evolution of complex higher brain centers and behaviors: behavioral correlates of mushroom body elaboration in insects. *Brain Behav. Evol.* 82 9–18. 10.1159/000352057 23979452

[B13] Farris S. M. Robinson G. E. Davis R. L. Fahrbach S. E. (1999). Larval and pupal development of the mushroom bodies in the honey bee, *Apis mellifera*. *J. Comp. Neurol.* 414 97–113. 10.1002/(SICI)1096-9861(19991108)414:1<97::AID-CNE8>3.0.CO;2-Q 10494081

[B14] Frisch B. Aschoff J. (1987). Circadian rhythms in honeybees: entrainment by feeding cycles. *Physiol. Entomol.* 12 41–49. 10.1111/j.1365-3032.1987.tb00722.x

[B15] Fuchikawa T. Beer K. Linke-Winnebeck C. Ben-David R. Kotowoy A. Tsang V. W. K. (2017). Neuronal circadian clock protein oscillations are similar in behaviourally rhythmic forager honeybees and in arrhythmic nurses. *Open Biol.* 7:170047. 10.1098/rsob.170047 28615472 PMC5493776

[B16] Giurfa M. (2007). Behavioral and neural analysis of associative learning in the honeybee: a taste from the magic well. *J. Comp. Physiol. A* 193 801–824. 10.1007/s00359-007-0235-9 17639413

[B17] Gould J. L. (1987). Honey bees store learned flower-landing behaviour according to time of day. *Anim. Behav.* 35 1579–1581. 10.1016/S0003-3472(87)80038-6

[B18] Gronenberg W. (2001). Subdivisions of hymenopteran mushroom body calyces by their afferent supply. *J. Comp. Neurol.* 436 474–489. 10.1002/cne.1045 11406827

[B19] Hourcade B. Muenz T. S. Sandoz J. C. Rössler W. Devaud J. M. (2010). Long-term memory leads to synaptic reorganization in the mushroom bodies: a memory trace in the insect brain? *J. Neurosci.* 30 6461–6465. 10.1523/JNEUROSCI.0841-10.2010 20445072 PMC6632731

[B20] Khamis A. M. Hamilton A. R. Medvedeva Y. A. Alam T. Alam I. Essack M. (2015). Insights into the transcriptional architecture of behavioral plasticity in the honey bee *Apis mellifera*. *Sci. Rep.* 5:11136. 10.1038/srep11136 26073445 PMC4466890

[B21] Koltermann R. (1971). 24-Std-periodik in der Langzeiterinnerung an Duft- und Farbsignalebei der Honigbiene. *Z. Vergl. Physiol.* 75 49–68. 10.1007/BF00335137

[B22] Lefer D. Perisse E. Hourcade B. Sandoz J. Devaud J. M. (2012). Two waves of transcription are required for long-term memory in the honeybee. *Learn. Mem.* 20 29–33. 10.1101/lm.026906.112 23247252

[B23] Lutz C. C. Robinson G. E. (2013). Activity-dependent gene expression in honey bee mushroom bodies in response to orientation flight. *J. Exp. Biol.* 216 2031–2038. 10.1242/jeb.084905 23678099 PMC3656508

[B24] McQuillan H. J. Nakagawa S. Mercer A. (2012). Mushroom bodies of the honeybee brain show cell population-specific plasticity in expression of amine-receptor genes. *Learn. Mem.* 19 151–158. 10.1101/lm.025353.111 22411422

[B25] Mistlberger R. (1994). Circadian food-anticipatory activity: formal models and physiological mechanisms. *Neurosci. Biobehav. Rev.* 18 171–195. 10.1016/0149-7634(94)90023-X 8058212

[B26] Mizunami M. Weibrecht J. M. Strausfeld N. J. (1998). Mushroom bodies of the cockroach: their participation in place memory. *J. Comp. Neurol.* 402 520–537. 10.1002/(SICI)1096-9861(19981228)402:4<520::AID-CNE6>3.0.CO;2-K 9862324

[B27] Moore D. (2001). Honey bee circadian clocks: behavioral control from individual workers to whole-colony rhythms. *J. Insect Physiol.* 47 843–857. 10.1016/S0022-1910(01)00057-9

[B28] Moore D. Doherty P. (2009). Acquisition of a time-memory in forager honey bees. *J. Comp. Physiol.* 195 741–751. 10.1007/s00359-009-0450-7 19462172

[B29] Moore D. Siegfried D. Wilson R. Rankin M. A. (1989). The influence of time of day on the foraging behavior of the honeybee. *Apis mellifera*. *J. Biol. Rhythm* 4 305–325. 10.1177/074873048900400301 2519596

[B30] Naeger N. L. Van Nest B. N. Johnson J. N. Boyd S. D. Southey B. R. Rodriguez-Zas S. L. (2011). Neurogenomic signatures of spatiotemporal memories in time-trained forager honey bees. *J. Exp. Biol.* 214 979–987. 10.1242/jeb.053421 21346126 PMC4074293

[B31] Pahl M. Zhu H. Pix W. Tautz J. Zhang S. (2007). Circadian timed episodic-like memory a bee knows what to do when, and also where. *J. Exp. Biol.* 210 3559–3567. 10.1242/jeb.005488 17921157

[B32] Prabhu C. Cheng K. (2008). One day is all it takes: circadian modulation of the retrieval of colour memories in honeybees. *Behav. Ecol. Sociobiol.* 63 11–22. 10.1007/s00265-008-0631-3

[B33] Renner M. (1955). Uber die Haltung von Bienen in geschlossenen, kunstlich beleuchteten Raumen. *Naturwissenschaften* 42 539–540. 10.1007/BF00630155

[B34] Renner M. (1957). NeueVersucheuber den Zeitsinn der Honigbiene. *Z. vergl. Physiol.* 40 85–118. 10.1007/BF00298152

[B35] Renner M. (1959). Uber ein weiters Versetzungsexperiment zur Analyse des Zeitsinnes und der Sonnenorientierung der Honigbiene. *Zeitschrift fur Vergleichende Physiologie* 42 449–483. 10.1007/BF00297804

[B36] Riessberger U. Crailsheim K. (1997). Short-term effect of different weather conditions upon the behaviour of forager and nurse honeybees (*Apis mellifera* carnica Pollmann). *Apidologie* 28 411–426. 10.1051/apido:19970608

[B37] R Core Team (2017). *R: A Language and Environment for Statistical Computing.* Vienna: R Foundation for Statistical Computing Available at: https://www.R-project.org/

[B38] Schröter U. Menzel R. (2003). A new ascending sensory tract to the calyces of the honeybee mushroom body, the subesophageal-calycal tract. *J. Comp. Neurol.* 465 168–178. 10.1002/cne.10843 12949779

[B39] Singh A. S. Shah A. Brockmann A. (2017). Honey bee foraging induces up-regulation of early growth response protein 1, hormone receptor 38 and candidate downstream genes of the ecdysteroid signalling pathway. *Insect Mol. Biol.* 27 90–98. 10.1016/j.febslet.2006.04.016 28987007

[B40] Sommerlandt F. M. Roessler W. Spaethe J. (2016). Impact of light and alarm pheromone on immediate early gene expression in the European honeybee, *Apis mellifera*. *Entomol. Sci.* 20 122–126. 10.1111/ens.12234

[B41] Strausfeld N. J. (2002). Organization of the honey bee mushroom body: representation of the calyx within the vertical and gamma lobes. *J. Comp. Neurol.* 450 4–33. 10.1002/cne.10285 12124764

[B42] Takeuchi H. Paul R. K. Matsuzaka E. Kubo T. (2007). EcR- A Expression in the brain and ovary of the honeybee (*Apis mellifera* L.). *Zoolog. Sci.* 24 596–603. 10.2108/zsj.24.596 17867861

[B43] von Frisch K. (1967). *Dance Language and Orientation of Bees.* Cambridge, MA: Harvard University Press.

[B44] Wagner A. E. Van Nest B. N. Hobbs C. Moore D. (2013). Persistence, reticence, and the management of multiple time memories by forager honey bees. *J. Exp. Biol.* 216 1131–1141. 10.1242/jeb.064881 23197093

[B45] Wahl O. (1932). NeueUntersuchungenüber das Zeitgedächtnis der Bienen. *Z. Vergl. Physiol.* 16 529–589. 10.1007/BF00338333

[B46] Wahl O. (1933). BeitragzurFrage der BiologischenBedeutung des Zeitgedächnisses der Bienen. *Z. Vergl. Physiol.* 18 709–717. 10.1007/BF00338365

[B47] Yamazaki Y. Shirai K. Paul R. K. Fujiyuki T. Wakamoto A. Takeuchi H. (2006). Differential expression of HR38 in the mushroom bodies of the honeybee brain depends on the caste and division of labor. *FEBS Lett.* 580 2667–2670. 10.1016/j.febslet.2006.04.016 16647071

[B48] Zhang S. Schwarz S. Pahl M. Zhu H. Tautz J. (2006). Honeybee memory: a honeybee knows what to do and when. *J. Exp. Biol.* 209 4420–4428. 10.1242/jeb.02522 17079712

