# Supplementary material for: Egr-1: A Candidate Transcription Factor Involved in Molecular Processes Underlying Time-Memory
Source: Front Psychol. 2018 Jun 5;9:865. doi: 10.3389/fpsyg.2018.00865 (PMC5997935; doi:10.3389/fpsyg.2018.00865)
Supplement: Supplementary file 1 [file Table_1.PDF]

Table S1: Adjusted p-values for No Food Reward Experiment (Ad libitum)

	10:00	14:00	18:00	22:00	02:00
14:00	0.18				
18:00	0.42	0.25			
22:00	**0.0161**	**0.0016**	**0.0095**		
02:00	**0.0415**	**0.0059**	**0.0295**	0.34	
06:00	**0.0474**	**0.0057**	**0.0334**	0.32	0.45
