# Supplementary material for: Egr-1: A Candidate Transcription Factor Involved in Molecular Processes Underlying Time-Memory
Source: Front Psychol. 2018 Jun 5;9:865. doi: 10.3389/fpsyg.2018.00865 (PMC5997935; doi:10.3389/fpsyg.2018.00865)
Supplement: Supplementary file 2 [file Table_2.PDF]

Table S2: Adjusted p-values for No Food Reward Experiment (08:00-10:00 trained)

| | 10:00 | 14:00 | 18:00 | 22:00 | 02:00 |
|---|---|---|---|---|---|
| 14:00 | 0.06 | | | | |
| 18:00 | **0.0002** | **0.0308** | | | |
| 22:00 | **0.0001** | **0.0197** | 0.36 | | |
| 02:00 | **0.0321** | 0.33 | 0.07 | **0.039** | |
| 06:00 | **0.0370** | 0.35 | 0.06 | 0.44 | **0.0326** |
