# Supplementary material for: Egr-1: A Candidate Transcription Factor Involved in Molecular Processes Underlying Time-Memory
Source: Front Psychol. 2018 Jun 5;9:865. doi: 10.3389/fpsyg.2018.00865 (PMC5997935; doi:10.3389/fpsyg.2018.00865)
Supplement: Supplementary file 3 [file Table_3.PDF]

Table S3: Adjusted p-values for No Food Reward Experiment(16:00-18:00 trained)

	10:00	14:00	18:00	22:00	02:00
14:00	0.33				
18:00	**0.0342**	0.07			
22:00	0.45	0.34	**0.0346**		
02:00	0.07	**0.0298**	**0.0002**	0.06	
06:00	0.08	**0.0407**	**0.0003**	0.45	0.07
