# Supplementary material for: Egr-1: A Candidate Transcription Factor Involved in Molecular Processes Underlying Time-Memory
Source: Front Psychol. 2018 Jun 5;9:865. doi: 10.3389/fpsyg.2018.00865 (PMC5997935; doi:10.3389/fpsyg.2018.00865)
Supplement: Supplementary file 4 [file Table_4.PDF]

Table S4: Adjusted p-values for 2-feeder Experiment (Single feeder visiting bees)

	E18:00	E09:00	M18:00
E09:00	**0.0083**		
M18:00	**0.0120**	0.37	
M09:00	0.43	**0.0069**	**0.0140**
