# Supplementary material for: Egr-1: A Candidate Transcription Factor Involved in Molecular Processes Underlying Time-Memory
Source: Front Psychol. 2018 Jun 5;9:865. doi: 10.3389/fpsyg.2018.00865 (PMC5997935; doi:10.3389/fpsyg.2018.00865)
Supplement: Supplementary file 5 [file Table_5.PDF]

Table S5: Adjusted p-values for 2-feeder Experiment (Both feeder visiting bees)

	13:00	18:00	22:00	02:00	06:00
18:00	**0.0147**				
22:00	0.38	**0.0075**			
02:00	0.37	**0.0063**	0.42		
06:00	0.48	**0.0109**	0.40	0.39	
09:00	**0.0305**	0.43	**0.0131**	**0.0101**	**0.0292**
