# Supplementary material for: Egr-1: A Candidate Transcription Factor Involved in Molecular Processes Underlying Time-Memory
Source: Front Psychol. 2018 Jun 5;9:865. doi: 10.3389/fpsyg.2018.00865 (PMC5997935; doi:10.3389/fpsyg.2018.00865)
Supplement: Supplementary file 6 [file Table_6.PDF]

Table S6: Adjusted p-values for 2-feeder Experiment (*Cry-2*)

	B18:00	B09:00	E18:00	E09:00	M18:00
B09:00	**0.0355**				
E18:00	0.47	**0.0305**			
E09:00	**0.0152**	0.36	**0.0127**		
M18:00	0.38	0.09	0.35	**0.0324**	
M09:00	0.47	**0.0302**	0.47	**0.0191**	0.35
