# Supplementary material for: Egr-1: A Candidate Transcription Factor Involved in Molecular Processes Underlying Time-Memory
Source: Front Psychol. 2018 Jun 5;9:865. doi: 10.3389/fpsyg.2018.00865 (PMC5997935; doi:10.3389/fpsyg.2018.00865)
Supplement: Supplementary file 7 [file Table_7.PDF]

Table S7: Adjusted p-values for Artificial Rain Experiment (16:00-18:00 trained, Pilot Experiment)

	06:00	10:00	14:00	15:00	15:30	16:00	16:30
10:00	0.30						
14:00	0.13	0.26					
15:00	**0.0013**	**0.0060**	**0.0347**				
15:30	**0.0326**	0.09	0.25	0.14			
16:00	**0.0082**	**0.0346**	0.14	0.27	0.31		
16:30	**0.0026**	**0.0079**	**0.0385**	0.50	0.15	0.28	
17:00	**0.0135**	**0.0388**	0.16	0.24	0.36	0.42	0.25
