# Supplementary material for: Egr-1: A Candidate Transcription Factor Involved in Molecular Processes Underlying Time-Memory
Source: Front Psychol. 2018 Jun 5;9:865. doi: 10.3389/fpsyg.2018.00865 (PMC5997935; doi:10.3389/fpsyg.2018.00865)

Figure S1. **(A)** Artificial Rain Set-up. **(B)** Pilot experiment to check *Egr-1* expression when the bees were prevented from flying out using “artificial set-up”. Bees were trained from 1600 to 1800 hours. The upregulation trend was initiated by 1400 hours, although not significant, which peaked at 1500 hours. Samples were collected till 1700 hours only to check for up-regulation in and around the training time. Data shown as relative expression changes compared to 0600 hours in the form of box-plots with individual data-points delineated , n=5. KW-test with Dunn’s(“bh” method) test to correct for multiple comparison done on each experiment, p-values in Table S7.

A

B

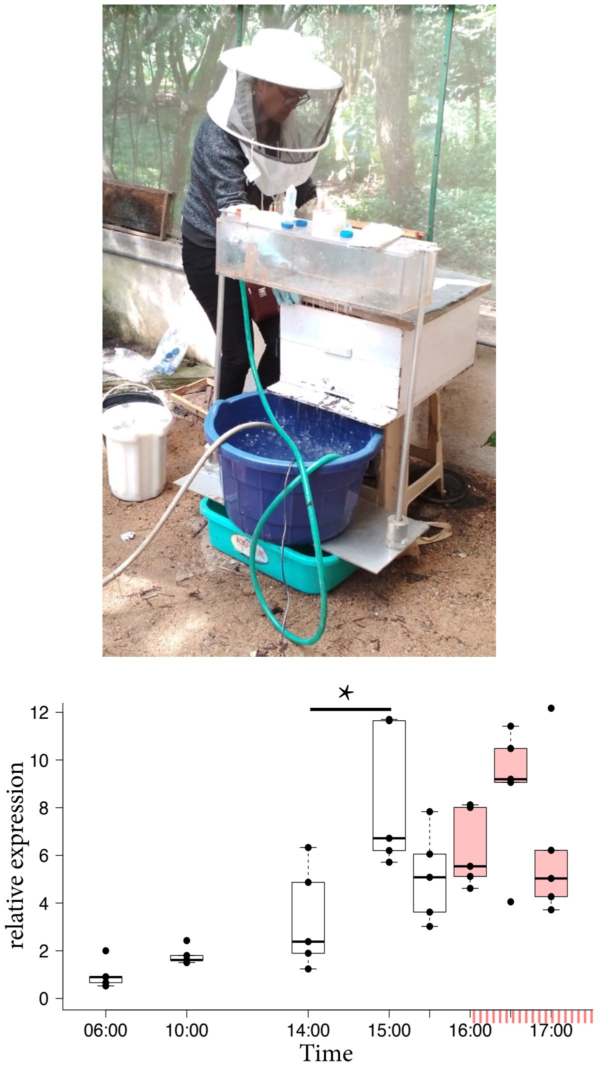

Supplement: Supplementary file 8 [file Image_1.PDF]
