# Supplementary material for: Egr-1: A Candidate Transcription Factor Involved in Molecular Processes Underlying Time-Memory
Source: Front Psychol. 2018 Jun 5;9:865. doi: 10.3389/fpsyg.2018.00865 (PMC5997935; doi:10.3389/fpsyg.2018.00865)

Figure S2: Behavioral observation of bee visits to the feeders in 2-feeder experiment. We recorded the feeder each hour for 3 minutes from 07:00 to 17:00 and counted the number of bees flying above the feeder and the number of landing attempts. For "flight", each count consists of every flight into and out of the frame of the video. For "land", each count consists of every land on the plate till the bee left the frame of the video. Since the bees were not individually marked, we cannot exclude counting single bee multiple times. Recordings were not made when the food reward was presented since bees are recruited at these times and hence is not a true representation of anticipation and search. The missing recordings have been marked with blue arrows (morning feeder open) and red arrows (evening feeder open). Both feeders were highly attractive during the time they were rewarded, but also were visited to a lesser amount when the other feeder was rewarded. However, most importantly both feeders were not visited during the hours when both did not provide any food reward.
(A) Recordings made at the morning feeder. **(B)** Recordings made at the evening feeder. Blue: Morning visits; Red: Evening visits; Black: In-between visits. Darker shade denotes "land". Lighter shade denotes "flight".

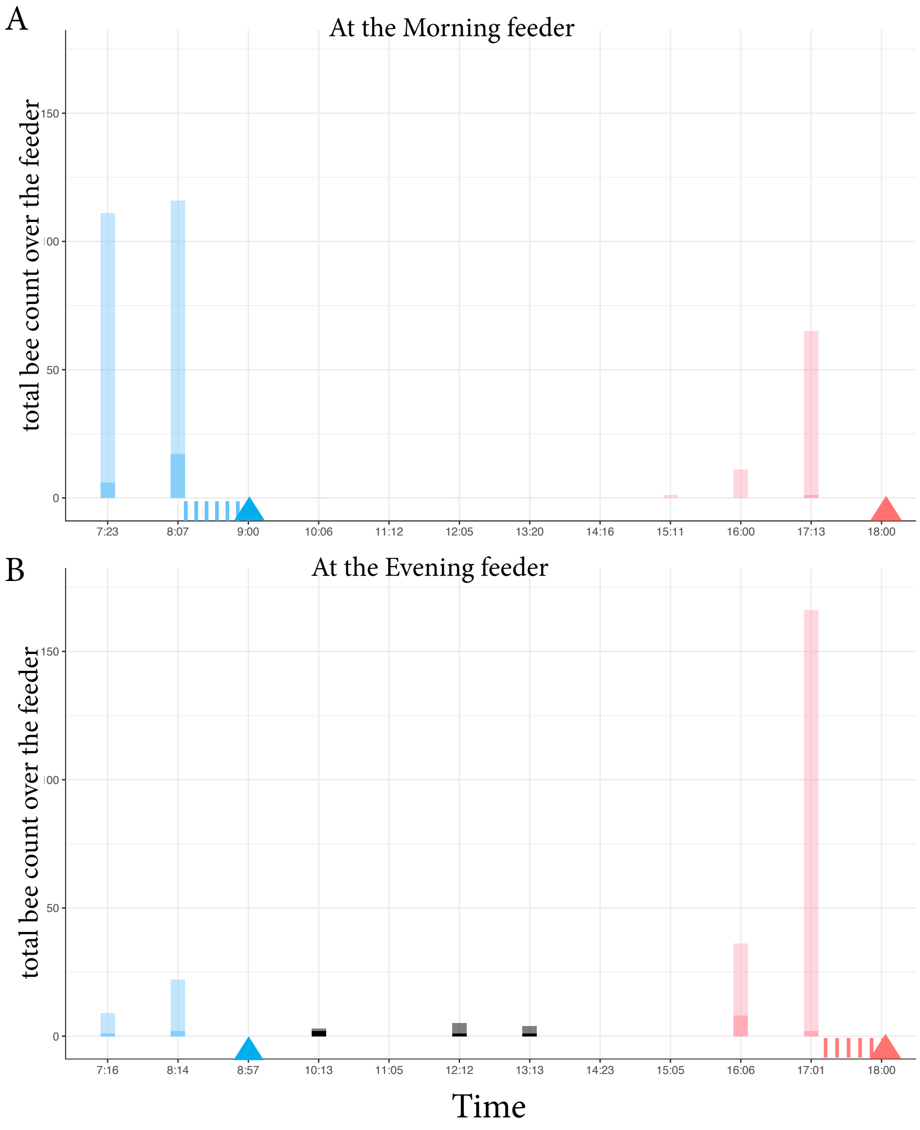

Supplement: Supplementary file 9 [file Image_2.PDF]
